# Spinal Cord Stimulation Frequency Influences the Hemodynamic Response in Patients with Disorders of Consciousness

**DOI:** 10.1007/s12264-018-0252-4

**Published:** 2018-07-11

**Authors:** Juanning Si, Yuanyuan Dang, Yujin Zhang, Yaxin Li, Wenyue Zhang, Yi Yang, Yue Cui, Xiaoping Lou, Jianghong He, Tianzi Jiang

**Affiliations:** 1 School of Instrumentation Science and Opto-electronics Engineering, Beijing Information Science and Technology University, Beijing, 100192 China; 2 Department of Neurosurgery, People’s Liberation Army General Hospital, Beijing, 100700 China; 3 Brainnetome Center, Institute of Automation, Chinese Academy of Sciences, Beijing, 100190 China; 4 National Laboratory of Pattern Recognition, Institute of Automation, Chinese Academy of Sciences, Beijing, 100190 China; 5 Key Laboratory for NeuroInformation of the Ministry of Education, School of Life Science and Technology, University of Electronic Science and Technology of China, Chengdu, 625014 China; 6 Chinese Academy of Sciences Center for Excellence in Brain Science, Institute of Automation, Chinese Academy of Sciences, Beijing, 100190 China; 7 Queensland Brain Institute, University of Queensland, St. Lucia, QL 4072 Australia

**Keywords:** Disorder of consciousness, Spinal cord stimulation, Frequency, Functional near-infrared spectroscopy, Hemodynamic response

## Abstract

Spinal cord stimulation (SCS) is a promising technique for treating disorders of consciousness (DOCs). However, differences in the spatio-temporal responsiveness of the brain under varied SCS parameters remain unclear. In this pilot study, functional near-infrared spectroscopy was used to measure the hemodynamic responses of 10 DOC patients to different SCS frequencies (5 Hz, 10 Hz, 50 Hz, 70 Hz, and 100 Hz). In the prefrontal cortex, a key area in consciousness circuits, we found significantly increased hemodynamic responses at 70 Hz and 100 Hz, and significantly different hemodynamic responses between 50 Hz and 70 Hz/100 Hz. In addition, the functional connectivity between prefrontal and occipital areas was significantly improved with SCS at 70 Hz. These results demonstrated that SCS modulates the hemodynamic responses and long-range connectivity in a frequency-specific manner (with 70 Hz apparently better), perhaps by improving the cerebral blood volume and information transmission through the reticular formation-thalamus-cortex pathway.

## Introduction

Advances in healthcare for brain injury have significantly increased the number of patients with chronic disorders of consciousness (DOCs) who survive brain injury [1]. However, there is no evidence-based treatment for DOC patients. The management of chronic DOCs is still challenging both for neuroscience and for clinical medicine [2].

Spinal cord stimulation (SCS) is a promising neuromodulation technique for the treatment of DOC patients. In SCS, electrodes implanted into the epidural space at C2–C4 deliver electrical impulses to stimulate the ascending reticular activating system and regulate the awareness circuit [3]. According to the literature, hypometabolism and impaired widespread network connectivity occur in brain circuits that are the neuronal substrates of consciousness in DOC patients [4–6]. It is well-established that the recovery of consciousness in DOC patients is correlated with the restoration of metabolism and the improvement of functional connectivity in the awareness circuits [4–6]. SCS has shown benefits in improving the cerebral blood perfusion and promoting the cortical neuroplasticity [2, 7] of DOC patients, with better effects in clinical improvement than traditional pharmacological and non-pharmacological treatments [8–10], especially for minimally conscious state (MCS) patients [11, 12]. For instance, Yamamoto *et al.* reported that SCS increases the cerebral blood flow in MCS patients by 22.2% during the SCS period compared to baseline [11].

The parameters of SCS are programmable, such as frequency, amplitude, and pulse width [3]. The effects of SCS are markedly affected by these parameters. For example, frequency, which is a most important determinant of SCS, influences how often a neuron fires in response to an external stimulus [13]. Different frequencies of SCS therapy might have different neurochemical effects [13]. Therefore, the design of the frequency parameter is, theoretically, a key step in SCS treatment. However, in practice, the SCS frequency used in the clinical environment is determined primarily based on the subjective experience of clinicians and behavioral assessment scales [6, 14]. So far, studies concerning the effects of SCS frequency on DOCs are fragmented and limited. Among these limited studies, the frequency varies from 5 to 200 Hz, among which some frequencies such as 70 Hz [10] and 100 Hz [15] have been found to improve the cerebral blood perfusion and produce acceptable behavioral enhancement in DOC patients after long-term clinical observation [2]. However, the differences in the spatio-temporal responsiveness at different SCS frequencies remain unknown, and it is unclear whether a frequency-specific effect exists. Therefore, there is a critical need to explore a rapid, quantitative method of evaluating differences in the spatio-temporal responsiveness of the brain at different SCS frequencies.

We have been investigating the influences of different SCS frequencies on the brain of DOC patients since the last two years, in order to clarify these issues. According to our previous EEG study, significantly altered relative power and synchronization in the frontal cortex of MCS patients occur after 5 Hz, 70 Hz, or 100 Hz SCS [16]. However, owing to the interference of the electrical impulses of SCS with the EEG, the data were recorded before and immediately after the SCS procedure rather than during the SCS. Therefore, the real-time spatio-temporal responsiveness of the brain at different SCS frequencies remained uncertain. Functional near-infrared spectroscopy (fNIRS) [17–19] is a non-invasive, portable technology that is not subject to interference from electrical stimulation [18], and can be used for longitudinal monitoring, making it uniquely suited for investigating the real-time spatio-temporal responsiveness of brain activity in DOC patients during the SCS procedure. The feasibility of fNIRS-SCS measurement for DOC patients was demonstrated in our previous study [14]. In that study, we investigated the effects of the interval parameter of SCS and found that a shorter interval was better for improving the cerebral blood volume during the SCS procedure.

Therefore, in this pilot study, we used a portable fNIRS system to measure the real-time hemodynamic responses over the prefrontal and occipital areas during the SCS procedure and investigated the effects of SCS at different frequencies on the spatio-temporal responsiveness of DOC patients.

## Materials and Methods

### Participants

**Fig. 1:**
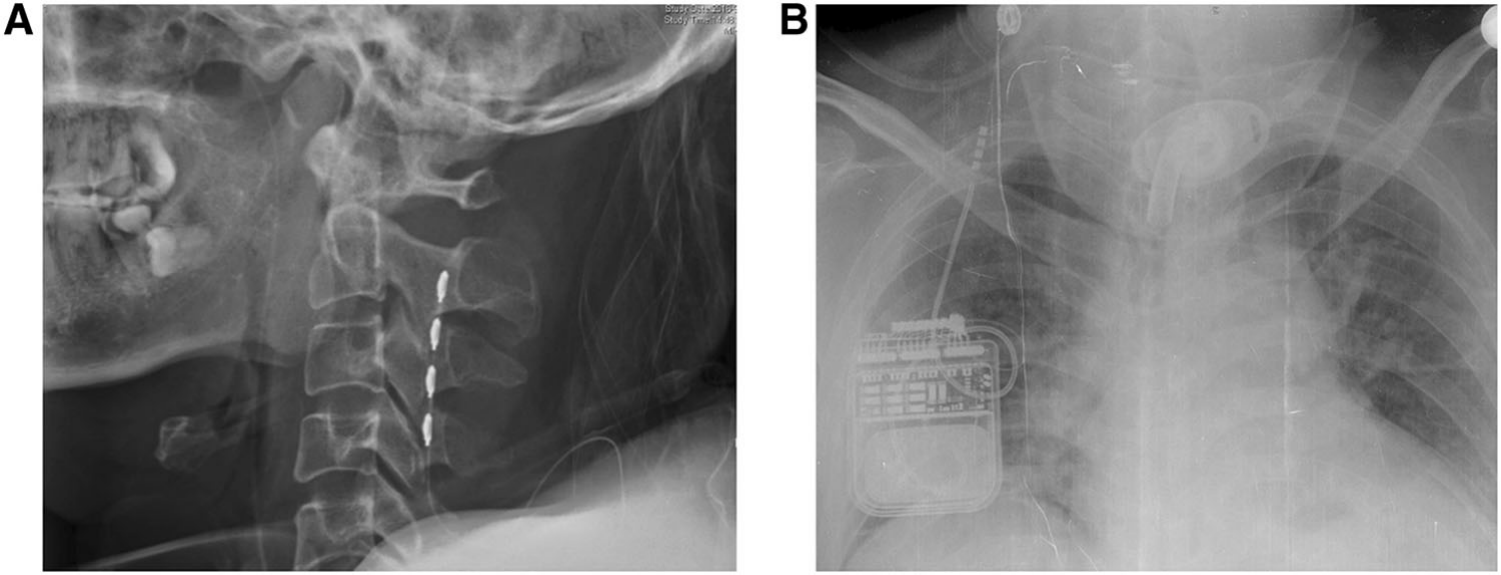
X-ray images illustrating the location of implanted SCS devices. **A** An SCS electrode implanted in the epidural space at C2–C4. **B** An implantable impulse generator arranged subcutaneously on the collarbone.

Ten DOC patients (5 males and 5 females, aged 17–64 years) were recruited from the Department of Neurosurgery, People’s Liberation Army General Hospital, Beijing, China. Each patient was implanted with an SCS device about 1 month before the study and was in a stable clinical state. An SCS electrode (3587A, Medtronic Inc., Minneapolis, MN) was implanted into the epidural space of the cervical vertebrae at the C2–C4 levels (Fig. 1). The corresponding implantable pulse generator was arranged subcutaneously on the collarbone. An SCS titration phase began 30 days after implantation. The clinical features of the DOC patients are listed in Table 1. The JFK Coma Recovery Scale was used to assess the state of consciousness of each DOC patient before SCS surgery [20]. Written informed consent was given by the patient’s caregivers. This study was approved by the Ethics Committee of the PLA Army General Hospital.

**Table 1:** Clinical features of patients with disorders of consciousness.

No.	Diagnosis	Gender	Age (years)	Duration of DOC (months)	Etiology	CRS-R
Patient 1	VS	M	17	4	Head trauma	6
Patient 2	VS	F	17	11	Head trauma	7
Patient 3	VS	M	18	8	Anoxic	6
Patient 4	MCS	F	29	28	Anoxic	9
Patient 5	VS	F	36	5	Anoxic	6
Patient 6	MCS	F	41	3	Anoxic	8
Patient 7	VS	M	42	6	Stroke	7
Patient 8	VS	M	53	12	Cerebral trauma	6
Patient 9	VS	M	54	11	Cerebral hemorrhage	8
Patient 10	VS	F	64	24	Cerebral hemorrhage	7

*VS* vegetative state; *MCS* minimally conscious state; *CRS-R* coma recovery scale-revised.

### Study Design

**Fig. 2:**
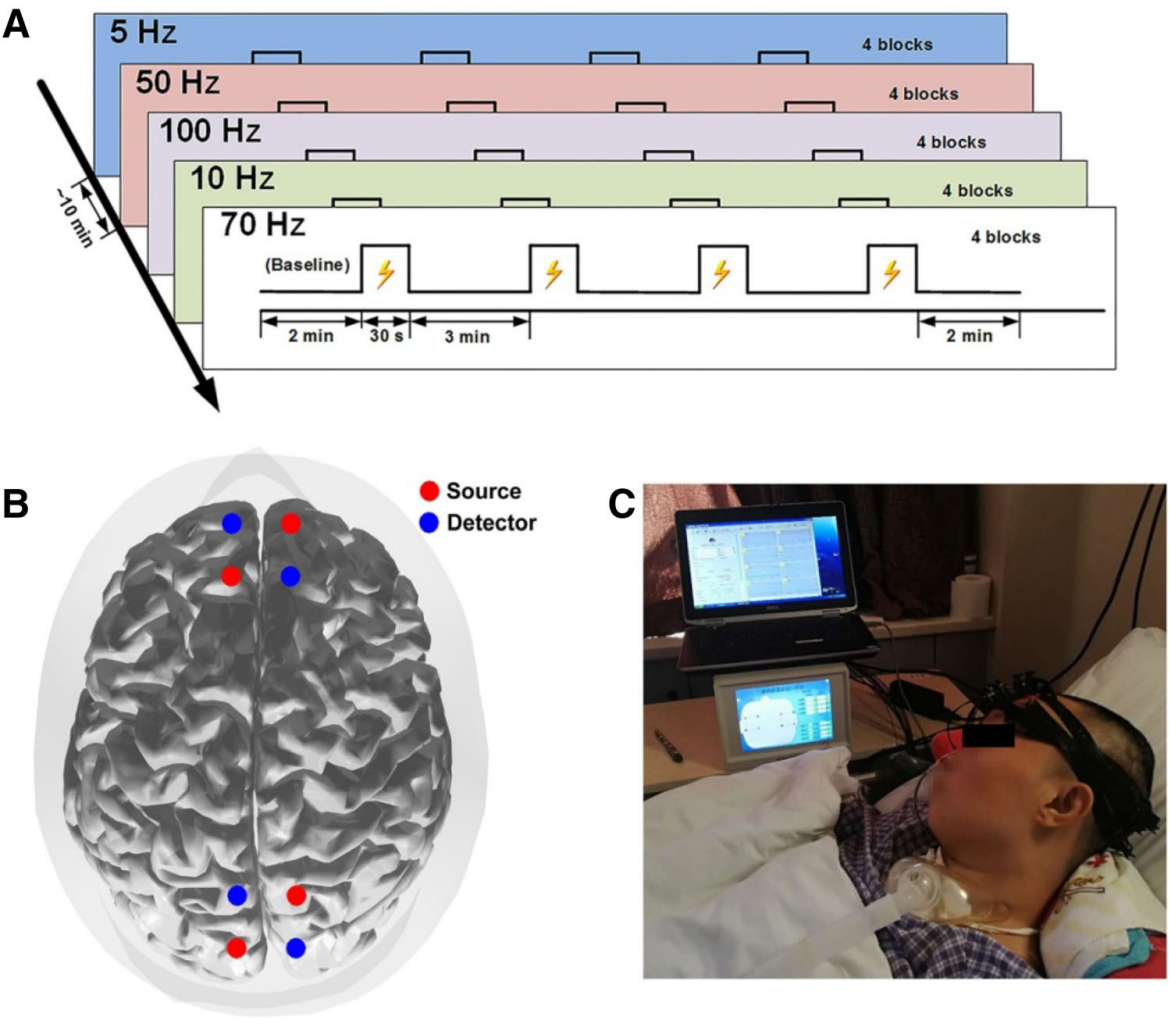
The experimental configuration. **A** Experimental paradigm for SCS. **B** Arrangement of probes over the prefrontal and occipital areas. **C** Photograph of the experimental setup.

The experimental paradigm was block-designed (Fig. 2A). The stimulation parameters for SCS were configured by a wireless controller. Specifically, the SCS parameters were as follows: frequency 5 Hz, 10 Hz, 50 Hz, 70 Hz, and 100 Hz; pulse width 210 μs; duration 30 s, and washout period 3 min, based on our previous study [14]. Because the neuronal and physiological sensitivity differed greatly among patients, we used different stimulation intensities (ranging from 1.0 V to 5.0 V, without reaching the motor threshold) for different DOC patients, individually determined by an experienced clinician. Each patient received five different frequencies at a fixed stimulation intensity. Each session of SCS consisted of four blocks. To control the overall duration of the experiment and to avoid the influence of the previous session to the next session, each patient was given a 10-min rest after each session. The five frequencies were presented in a pseudo-random order. Each DOC patient received SCS on three different days with different sequences of frequencies.

### fNIRS Recording

The hemodynamic responses of the DOC patients were recorded using a custom-designed portable fNIRS system [21]. Two pads covered with optodes were placed on the scalp above the prefrontal and occipital areas. Each pad consisted of two sources and two detectors, yielding 4 optical channels (Fig. 2B). The distance between the source and detector pairs was 3 cm, and each pad covered an area of ~ 3 × 3 cm^2^. The sampling rate of the fNIRS was 100 Hz.

### Data Analysis

Data were processed using the MatLab 2013a platform (MathWorks Inc., Natick, MA). First, the relative concentration changes of oxygenated, deoxygenated, and total (HbT) hemoglobin were calculated based on the modified Beer-Lambert Law [22, 23]. Since the HbT concentration is proportional to changes in regional cerebral blood volume [24, 25], we specified the hemodynamic responses mainly on the basis of the HbT concentration. Second, the hemodynamic data were low-pass filtered at 0.3 Hz to remove task-unrelated noise. Then the data were segmented into several epochs, starting 30 s before the SCS onset and ending 90 s after the stimulation, and epochs with large artifacts were rejected. After baseline correction (calculated from the 30-s epoch before SCS onset), the block-averaged hemodynamic responses were calculated. Next, the block-averaged hemodynamic responses were averaged across the channels over the prefrontal and occipital areas, separately. Finally, the group-averaged hemodynamic responses were calculated.

The functional connectivity was evaluated by calculating the correlation values of the time courses of the hemodynamic responses between the prefrontal and occipital areas for the five frequencies. First, the whole time courses of the hemodynamic responses were averaged across the four channels over the prefrontal and occipital areas, separately. Then, the time courses of the 80 s before the SCS onset were extracted as segmentations for further calculation. Next, the correlation values of the segmentations between the prefrontal and occipital areas were calculated. The first correlation value indicated the functional connectivity before the SCS procedure, whereas the averaged correlations for the remaining segmentations presented the functional connectivity after the SCS procedure.

All data are presented as mean ± standard error unless otherwise stated. The *t*-test and one-way analysis of variance (ANOVA) were used to characterize the differences between different conditions. For *post hoc* analysis, the least-significant difference (LSD) algorithm was used. Differences were accepted as significant when *P* < 0.05.

## Results

**Fig. 3:**
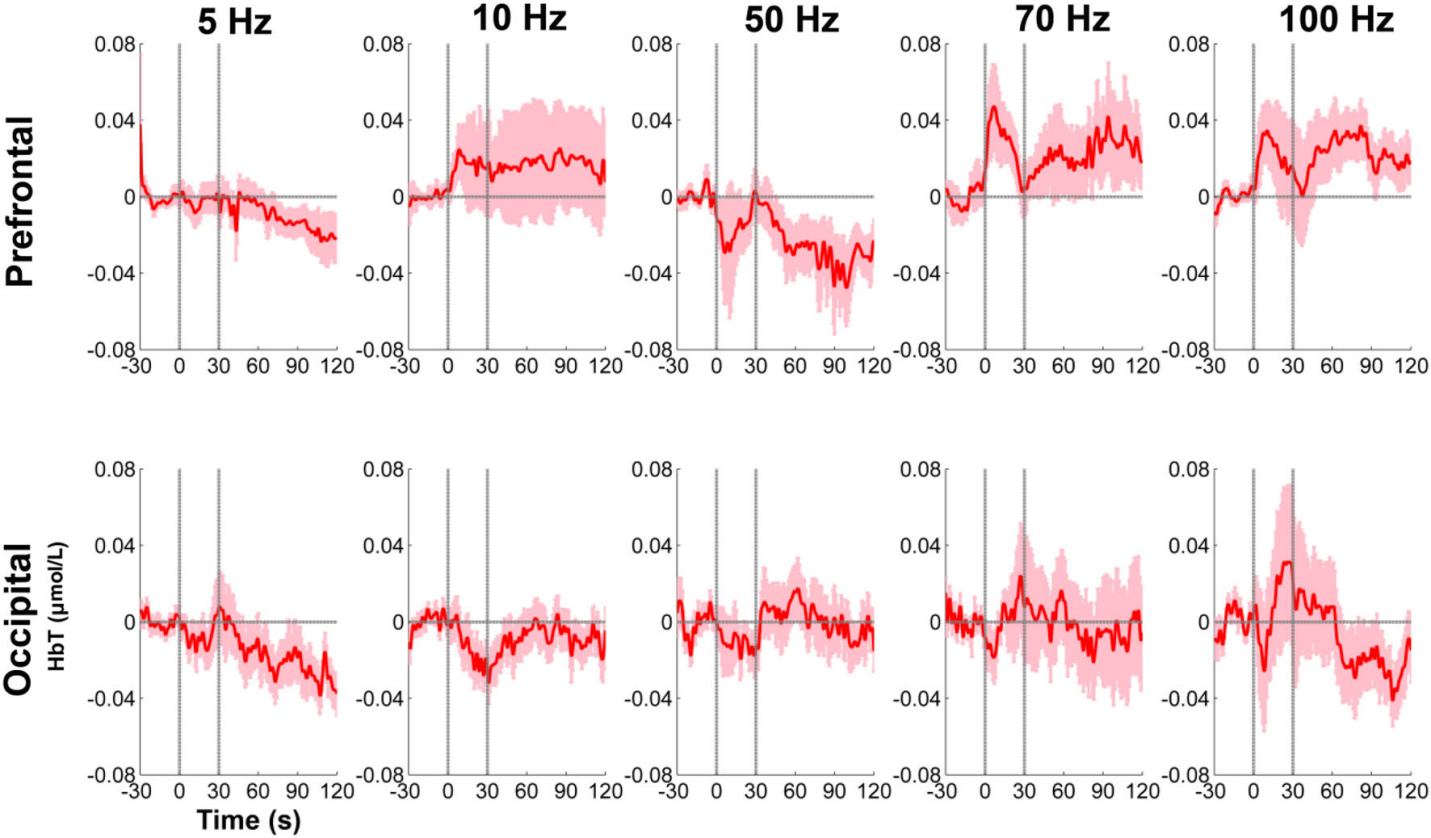
Group-averaged hemodynamic responses of DOC patients during the SCS procedure in the prefrontal and occipital areas. The SCS duration is indicated by the space between the two vertical gray lines. The shadowed areas indicate the standard errors of the mean values across all patients.

During the baseline (pre-SCS) period, the HbT concentrations over the prefrontal and occipital areas at all five frequencies were relatively stable (Fig. 3). At SCS onset, the HbT concentrations over the prefrontal and occipital areas at different frequencies changed in different ways. Specifically, at the prefrontal area, the HbT concentration was significantly higher during the on-SCS period of the 70 Hz SCS at the group level in terms of positive peak (*P* = 0.026) and mean (*P* = 0.039) values. However, there were no significant changes during the on-SCS period at the other SCS frequencies. After the SCS was turned off (post-SCS), the mean HbT concentration at 100 Hz was significantly different from baseline at the group level (*P* = 0.031). As for the occipital area, there were no significant changes at any frequencies at the group level.

**Fig. 4:**
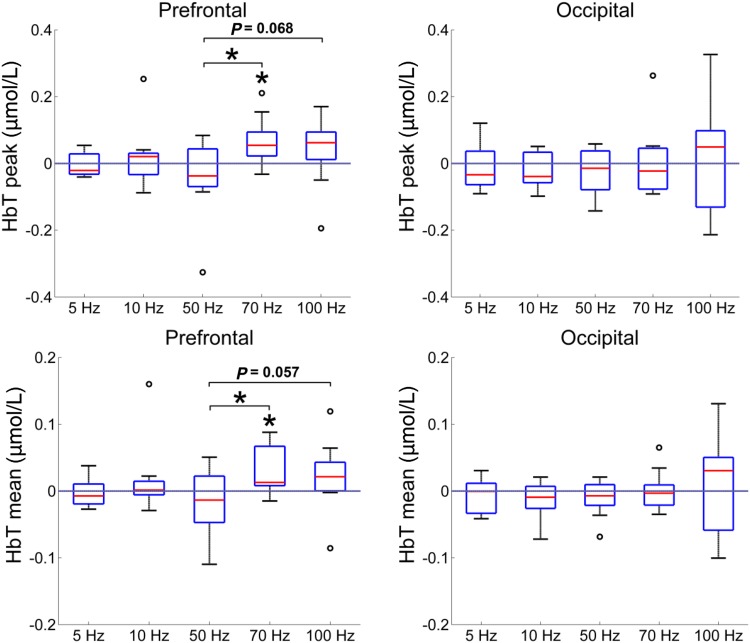
Peak and mean values of the HbT concentrations among the five SCS frequencies during the on-SCS period. Error bars indicate the standard errors of the mean (*P < 0.05).

To further explore the differences between the SCS-evoked hemodynamic fluctuations at the different frequencies, the peak and mean values of the hemodynamic responses during the on-SCS period were compared among the five frequencies using one-way ANOVA (Fig. 4). In the prefrontal area, although the peak (*F*(4,45) = 1.904, *P* = 0.126) and the mean (*F*(4,45) = 1.769, *P* = 0.152) values did not significantly differ among the five frequencies, the values at 70 Hz were significantly higher than those at 50 Hz (peak value: *P* = 0.014, LSD corrected; mean value: *P* = 0.028, LSD corrected). In the occipital area, there were no significant differences in either the peak or the mean values between any frequencies at the group level.

In addition, the mean HbT values during the post-SCS period showed a trend of significant difference among the five frequencies (*F*(4,45) = 2.228, *P* = 0.081) in the prefrontal area. Specifically, there were significant differences between 50 Hz and 70 Hz (*P* = 0.039, LSD corrected), and between 50 Hz and 100 Hz (*P* = 0.015, LSD corrected). This finding was consistent with that during the on-SCS period. As for the occipital cortex, there were no significant differences between any pair-wise conditions.

**Fig. 5:**
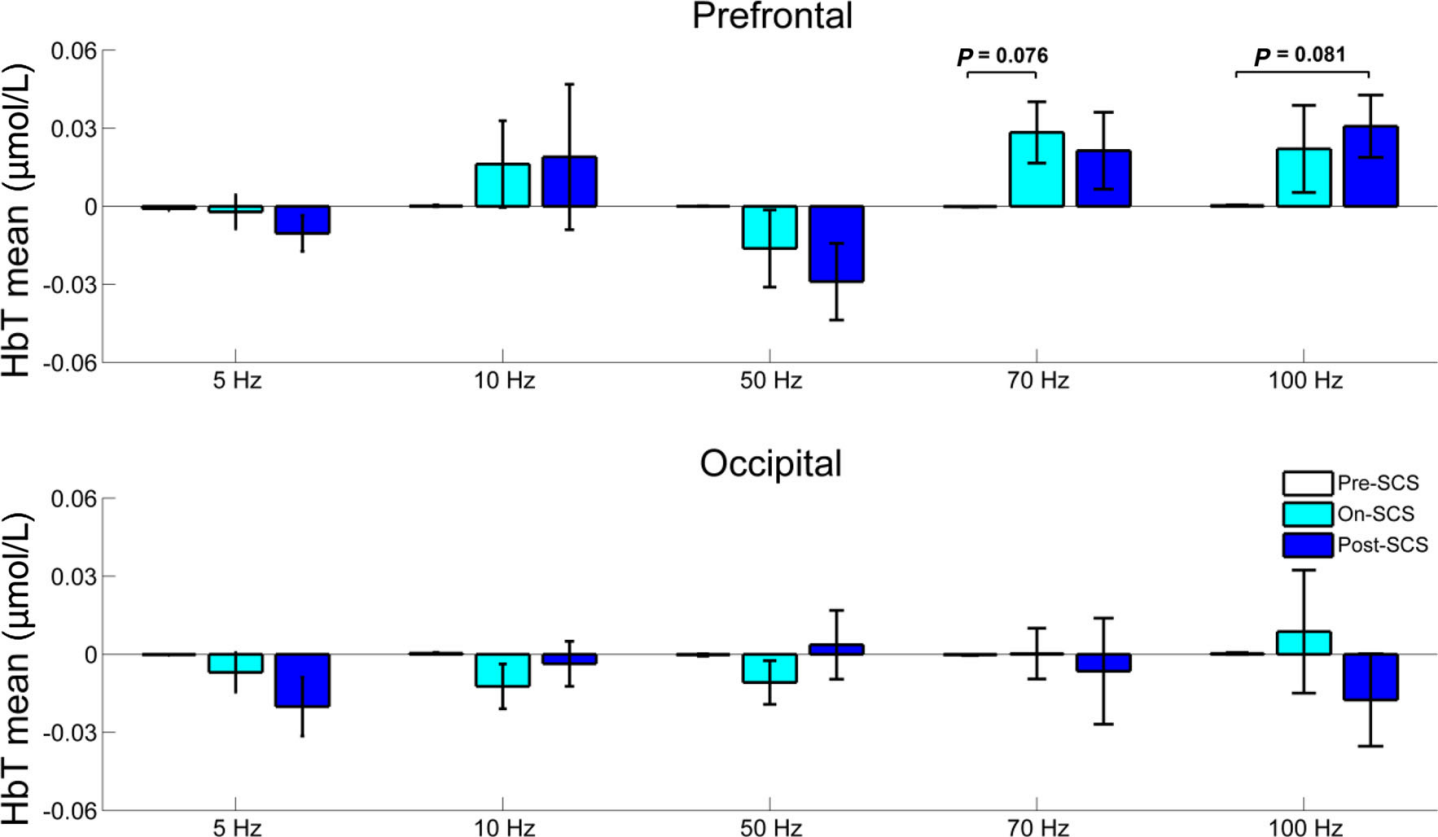
Mean HbT concentrations at the five different SCS frequencies in the pre-, on-, and post-SCS periods. Error bars indicate the standard errors of the mean (*P < 0.05).

We further analyzed the short-term dynamic changes of the hemodynamic responses to stimulation at different frequencies, specifically by comparing the mean values of HbT concentrations in the pre-SCS, on-SCS, and post-SCS periods using one-way ANOVA (Fig. 5). At the prefrontal cortex, although there was no significant difference among the three periods with 70-Hz SCS (*F*(2,27) = 1.855, *P* = 0.176), the difference between the on-SCS and pre-SCS periods still trended toward significant (*P* = 0.076, LSD corrected). Similarly, at 100 Hz SCS, the difference between the post-SCS and pre-SCS periods trended toward significance (*P* = 0.081, LSD corrected). At the other frequencies, pair-wise comparisons did not show any significant differences among the three periods.

**Fig. 6:**
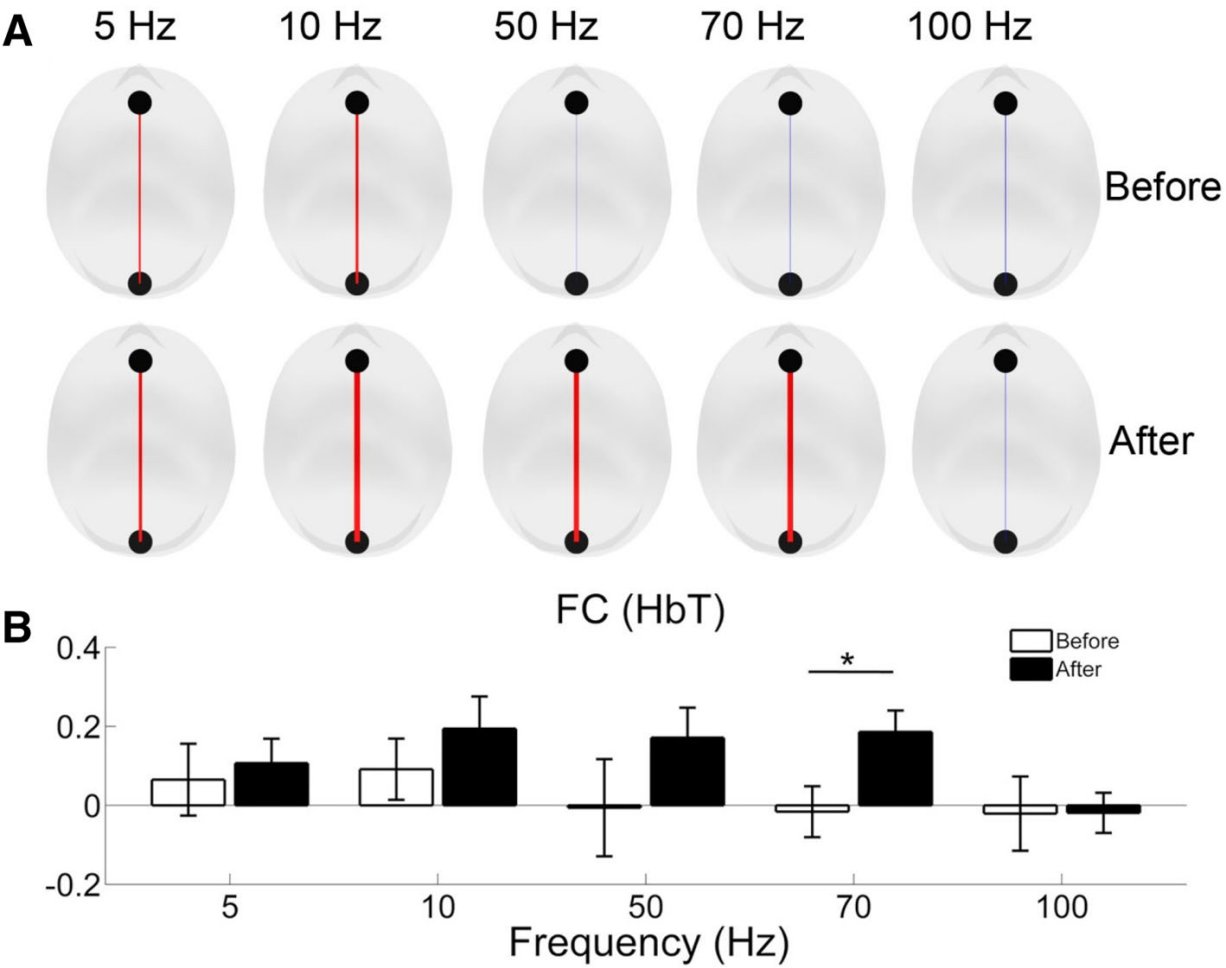
Effects of SCS on the functional integrity of the brain. **A** Group-level results of functional connectivity between the prefrontal and occipital areas for the five frequencies in the period before and after the SCS procedure. Red lines indicate positive, and blue lines negative correlation values. **B** Group-level functional connectivity between the periods before and after the SCS procedure for the five frequencies. White and black bars represent the means of the functional connectivity coefficients before and after the SCS procedure, respectively. Error bars indicate the standard error of the mean (*P < 0.05).

To further explore the effects of SCS on the functional integrity of the brain, we calculated the functional connectivity using ROI-based correlation analysis between the prefrontal and occipital areas. At 5, 10, 50, and 70 Hz, there were clear increases in functional connectivity after the SCS procedure compared to the period before SCS onset, but the increase was statistically significant only at 70 Hz (*P* = 0.01) (Fig. 6). At the individual level, 70-Hz SCS induced functional connectivity improvement in 80% (8/10) of the DOC patients. By combining the hemodynamic responses and the functional connectivity, 50% (4/8) of those with improved functional connectivity after 70-Hz SCS also showed the largest increases of the hemodynamic response with 70-Hz SCS.

## Discussion

The effective utility of SCS in the treatment for DOC patients necessitates an understanding of the effects of different SCS parameters on the brain. In this pilot study, we used fNIRS to measure the hemodynamic changes in real time over the prefrontal and occipital areas of DOC patients during the SCS procedure. The differences in cerebral hemodynamic responses and functional connectivity were evaluated to investigate the effects of SCS at different frequencies in DOC patients.

### Effects of Different SCS Frequencies on Hemodynamic Responses

The distributions of the SCS-evoked hemodynamic responses of the five frequencies (5 Hz, 10 Hz, 50 Hz, 70 Hz, and 100 Hz) were clearly distinct, implying that the SCS frequency modulates the spatio-temporal responsiveness of brain activity in DOC patients. Specifically, at 70 Hz, we found significant increases in the peak values, as well as the mean values, of the HbT concentration in the prefrontal cortex during the SCS procedure. Significant increases in the mean values of the hemodynamic responses were also found in the post-SCS period at 100 Hz. This implies that SCS at 70 Hz and 100 Hz improves the cerebral blood volume and metabolism in DOC patients, and the SCS-evoked cerebral blood volume changes occur earlier at 70 Hz than at 100 Hz. Interestingly, both the mean and the peak values of the SCS-evoked hemodynamic responses during 50-Hz stimulation in the prefrontal cortex were significantly (trended toward significantly) different from those at 70 Hz (100 Hz), implying that the underlying effects of 50-Hz SCS might differ from those at 70 Hz and 100 Hz.

Above all, these findings showed that SCS modulates the hemodynamic responses of DOC patients in a frequency-specific manner. We suggest that the frequency-specific effects of SCS might be due to the different neurochemical effects induced by SCS. For example, as reported by Miller *et al.*, when applying SCS through implants in the dorsal spinal cord for the management of neuropathic pain, the stimulation frequency is a key determinant for activating different neurochemical mechanisms. Specifically, low frequencies (2 Hz–10 Hz) activate μ-opioid receptor pathways, whereas high frequencies (~ 100 Hz) activate endogenous δ-opioid receptor pathways. Frequencies ~ 50 Hz activate dorsal horn GABAergic neurons [26]. However, because of the different target areas in which the stimulator was implanted and the different pathological mechanisms underlying neuropathic pain and DOCs, whether such neurochemical effects of SCS on neuropathic pain also work on DOCs remains unknown and needs to be further explored.

### Effects of Different SCS Frequencies on Functional Connectivity

It has been reported that DOCs might not only be associated with hypometabolism in widespread networks, but also with significantly decreased functional connectivity in the whole brain, especially long-distance functional connectivity [5, 27–29]. Interestingly, a study on DOC patients who regained consciousness showed functional restoration of metabolism and increased functional connectivity between the prefrontal cortex, thalamus, and other brain areas, and provided an important causal link between the functional integrity of the brain and consciousness [4, 29–32]. In our study, one kind of long-distance connectivity – functional connectivity between the prefrontal and occipital areas – significantly increased during 70-Hz SCS at the group level. At the individual level, 70-Hz SCS improved the functional connectivity for 80% (8/10) of DOC patients. Among them, 50% (4/8) also showed the largest increases of the hemodynamic response with 70-Hz SCS. These findings provide insights that SCS not only modulates the cerebral blood volume of DOC patients in a frequency-specific manner, but also improves the between-network connectivity (at least that between the prefrontal and occipital areas) at a specific frequency.

### Possible Mechanism Underlying SCS for the Treatment of DOC Patients

According to the mesocircuit model, in severe brain injuries, broad deafferentation and disconnection in the cortico-striatopallidal-thalamocortical loop results in a significant loss of input to the medium spiny neurons of the striatum, preventing these neurons from reaching their firing threshold [6]. Based on our findings, we suggest that a possible mechanism underlying SCS can be (at least partially) explained as follows: electrical modulation by SCS stimulates the ascending reticular formation-thalamus-cortex pathway along with effects associated with improvements in cerebral blood volume and long-range functional connectivity of the brain circuits crucial for maintaining consciousness in the “mesocircuit” model [6, 33]. The frequency-specific effects of SCS, especially the significant improvement of cerebral blood volume and long-distance functional connectivity at 70 Hz, also explain the possible mechanism underlying good behavioral enhancement in DOC patients by 70-Hz SCS [2, 10].

In addition, a significantly increased hemodynamic response in the prefrontal cortex and significantly improved prefrontal-occipital connectivity in DOC patients, indicate that the prefrontal cortex plays an important role in the effects of SCS on the cerebral cortex. Actually, it is well-established that the prefrontal cortex is one of the most important components underlying awareness [4, 6, 33]. First, studies in normal individuals examining conscious and non-conscious processing conditions have reported that prefrontal and parietal networks are associated with conscious access [34]. Second, transcranial magnetic stimulation over the prefrontal cortex can interrupt conscious perception and even disrupt conscious vision in normal individuals, also providing evidence that the prefrontal network plays a causal role in conscious perception [19, 35]. Third, in pathological brain states, positron emission tomography studies have found hypometabolism and dysfunctional damage in the thalamus, prefrontal cortex, and frontoparietal association areas in DOC patients [27, 30, 36]. In the “mesocircuit” model, the frontal/prefrontal cortico-striatopallidal-thalamocortical loop system has been proposed to be crucial for maintaining consciousness [33]. In the current study, the 70-Hz SCS-evoked hemodynamic responses in the prefrontal cortex were similar to those in typical tasks (such as working memory [37] and attention [38]). Moreover, based on a previous EEG study by our group, 70-Hz SCS significantly alters the relative power and synchrony of the delta and gamma bands in the frontal area rather than other areas [16]. Therefore, we infer that the findings on the prefrontal cortex in this study may show its functional significance in the recovery of consciousness in DOC patients.

## Limitations of the Current Study

Given that SCS is a newly-emerging neuromodulation technique for the treatment of DOC patients, the limited sample size and the wide patient-selection criteria are the main limitations of this pilot study. We hope that, with the development of neuromodulation techniques and the accumulation of patients among multiple centers, the current results will be confirmed and the specific effects of SCS parameters for DOC patients will be fully investigated. In addition, there is still a lack of long-term monitoring during routine SCS treatment or long-term clinical follow-up. Therefore, further investigation is needed on the relationship between the clinical effects and the frequency-specific effects on hemodynamic responses and functional connectivity. As the first study to investigate the hemodynamic and functional connectivity effects of SCS on the brains of DOC patients, it is clear that our current results are just the tip of the iceberg. We hope that our findings with significant frequency-specific effects provide some useful information for clinicians to design reasonable and effective SCS programming parameters for DOC patients. And since fNIRS is cost-effective, portable, and ecological and can be used for longitudinal monitoring, we hope it can be used to obtain new insights into brain function in this challenging patient population.
